# Spf1 and Ste24: quality controllers of transmembrane protein topology in the eukaryotic cell

**DOI:** 10.3389/fcell.2023.1220441

**Published:** 2023-08-03

**Authors:** Donald J. Tipper, Carol A. Harley

**Affiliations:** ^1^ University of Massachusetts Medical School, Worcester, MA, United States; ^2^ i3S-Instituto de Investigação e Inovação em Saude, Universidade do Porto, Porto, Portugal; ^3^ IBMC-Instituto de Biologia Molecular e Celular, Universidade do Porto, Porto, Portugal

**Keywords:** transmembrane proteins, topology, positive inside rule, quality control, topology error recognition

## Abstract

DNA replication, transcription, and translation in eukaryotic cells occur with decreasing but still high fidelity. In contrast, for the estimated 33% of the human proteome that is inserted as transmembrane (TM) proteins, insertion with a non-functional inverted topology is frequent. Correct topology is essential for function and trafficking to appropriate cellular compartments and is controlled principally by responses to charged residues within 15 residues of the inserted TM domain (TMD); the flank with the higher positive charge remains in the cytosol (inside), following the positive inside rule (PIR). Yeast (*Saccharomyces cerevisiae*) mutants that increase insertion contrary to the PIR were selected. Mutants with strong phenotypes were found only in *SPF1* and *STE24* (human cell orthologs are *ATP13A1* and *ZMPSte24*) with, at the time, no known relevant functions. Spf1/Atp13A1 is now known to dislocate to the cytosol TM proteins inserted contrary to the PIR, allowing energy-conserving reinsertion. We hypothesize that Spf1 and Ste24 both recognize the short, positively charged ER luminal peptides of TM proteins inserted contrary to the PIR, accepting these peptides into their large membrane-spanning, water-filled cavities through interaction with their many interior surface negative charges. While entry was demonstrated for Spf1, no published evidence directly demonstrates substrate entry to the Ste24 cavity, internal access to its zinc metalloprotease (ZMP) site, or active withdrawal of fragments, which may be essential for function. Spf1 and Ste24 comprise a PIR quality control system that is conserved in all eukaryotes and presumably evolved in prokaryotic progenitors as they gained differentiated membrane functions. About 75% of the PIR is imposed by this quality control system, which joins the UPR, ERAD, and autophagy (ER-phagy) in coordinated, overlapping quality control of ER protein function.

## Introduction

Essential background to transmembrane (TM) protein insertion mechanisms can be found in reviews (von Heijne, 2006; Shao and Hegde, 2011; Aviram et al., 2017; Lewis and Hegde, 2021; Dederer and Lemberg, 2021; Hegde and Keenan, 2022) and in recent publications (McKenna et al., 2020; McKenna et al., 2022; Smalinskaite et al., 2022). A review of algorithms for predicting TM protein topology from sequence profiles was published by Jones (2007). Alphafold (Heaven, 2020) predicts TM protein structures well (Hegedus et al., 2022) but may not yet consider the effects of membrane lipid composition. A recent review (Chen et al., 2023) describes the multiple quality control systems that recognize misfolded and aggregated endoplasmic reticulum (ER) luminal proteins: the unfolded protein response (UPR) comprises ER lumen–nucleus signaling mechanisms and re-export mechanisms; the ER-associated protein degradation (ERAD) system uses multiple ER membrane ubiquitin ligases to remove proteins that escape the UPR; and the ER-associated autophagy (ER-phagy) system delivers ERAD-resistant and aggregated ER proteins to the lysosome for destruction. The proposed PIR quality control system is potentially an important additional component.

## TM protein insertion, topology, functionality, and trafficking

The overall turnover of nascent proteins in mammalian cells is estimated at 30% (Schubert et al., 2000) and, given the error-prone nature of TM protein insertion, the turnover of this 33% of the proteome may exceed this average, so one-third or more of discarded nascent proteins, about 10% of the proteome, is fodder for error correction by ERAD and, principally, by Spf1/Ste24-mediated PIR quality control. The first stage of nuclear-encoded TM protein insertion is co-translational or post-translational targeting to the ER, the outside mitochondrial membrane (OMM), chloroplasts and other plastids, or peroxisomes (Aviram et al., 2017; McKenna et al., 2020; McKenna et al., 2022; Dahan et al., 2022; Hegde and Keenan, 2022). TM protein functions are dependent on the insertion of their hydrophobic TMDs in the ER with the correct topology, which is necessary for trafficking to their cellular sites of function, where they become markers for organelle recognition. ER insertion requires cooperation between cytoplasmic chaperones preventing TMD aggregation, such as the signal recognition particle, SRP (Walter and Blobel, 1981a; Walter and Blobel, 1981b; Akopian et al., 2013), ER receptors for these chaperones, and the TM protein complexes that mediate protein insertion and secretion. The evolution of these sorting components commenced in prokaryotes before the separation of bacteria and Archaea (Lewis et al., 2021; Hegde and Keenan, 2022). Intramembrane folding and assembly of TMDs of multiple TM proteins, or within proteins with multiple TMDs (multi-pass), is required to produce functional TM complexes (Hegde and Keenan, 2022; Smalinskaite et al., 2022). Schlebach and Sanders (2014) surveyed 470 known pathogenic mutations in five misfolding-prone TM proteins; about 10% had predicted and likely pathogenic effects on topogenesis.

## Topology and folding of TM proteins

**FIGURE 1:**
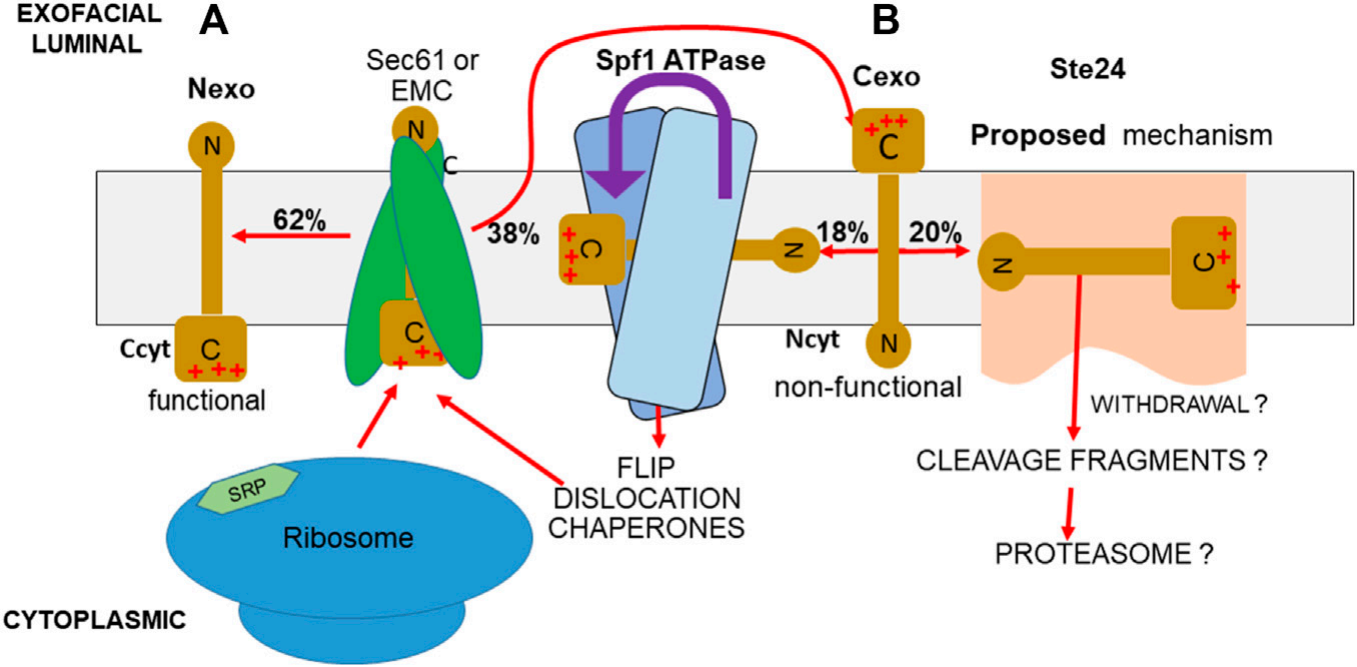
PIR quality control system. **(A)** If the more positively charged TMD-flanking peptide of a TM protein is the C-terminus, as in SPßla and SPInv fusions, the PIR requires that this flank remains in the cytoplasm (Ccyt/Nexo), in the functional orientation during insertion. **(B)** Any of this TM protein inserted with inverted topology (Ncyt/Cexo) is non-functional. Approximately half of this inverted protein is accepted by the Spf1 cavity for ATPase-driven dislocation, allowing reinsertion (McKenna et al., 2020; McKenna et al., 2022; center). Most of the rest is eliminated by Ste24. A hypothetical mechanism is the charge-directed entry into the Ste24 cavity and ZMP cleavage, possibly aided by removal by the Dfm1-recruited cdc48 AAA ATPase, followed by proteasomal disassembly (right).

The most N-terminal TMD of a TM protein (TMD1) can insert either Ncyt with the N-terminus remaining in the cytoplasm (Rapoport et al., 2017), or Nexo, with the N-terminus inserted into the lumen of the ER. These terms are pictorially defined in Figure 1. Orientation depends on responses to topogenic signals, which are principally provided by charged residues within 15 residues of the TMD (von Heijne, 1986). The charge difference spanning the TMD (C-terminal charges minus N-terminal charges) is the determinant of the positive inside rule (PIR) (von Heijne, 1986; von Heijne, 2006; Hartmann et al., 1989; Krogh et al., 2001). *In vivo* and *in vitro* studies in bacteria with genetically modified phospholipid composition demonstrated the role of local membrane composition in topogenesis (Vitrac et al., 2013). Local membrane surface charge, principally the ratio of phosphatidylserine (negative charge) to phosphatidylethanolamine (uncharged), can affect the influence of negatively peptide charges on the PIR (White and von Heijne, 2005; Vitrac et al., 2013; Baker et al., 2017), potentially allowing topological inversion on transit to a different membrane environment. Variation can lead to dual topologies, as for diacylglycerol transferase in HepG2 cells (Wurie et al., 2011). Other TM proteins are known to have dual topology (Rapp et al., 2006). N-glycosylation can also affect topology after insertion (Goder et al., 1999). These influences on topology are incorporated into The Charge Balance Rule (Dowhan et al., 2019), a more general version of the PIR that gives equal weight to positive and negative charges while allowing for the effects of the lipid environment.

The PIR/Charge Balance rule states that insertion should leave the more positively charged TMD-flanking peptide in the cytosol. The charge difference determines the strength of the bias (Harley and Tipper, 1996; Harley et al., 1998). The PIR was deduced from empirical observations in bacteria (von Heijne, 1986), where, as in Archaea, the membrane’s positive outside potential gradient provides a clear rationale. The complexity of TM protein insertion, topology determination, and effects on the topology of intramembrane complex assembly were reviewed by Shao and Hegde (2011). The hydrophobicity of the TMD modulates the response to the PIR flanking charges, and this effect is predicted by an algorithm based on amino acid insertion-energy profiles (Elazar et al., 2016). A recent review, “Folding and Misfolding of Human Membrane Proteins in Health and Disease” discussed the effects of the thermodynamics and kinetics of TM protein folding on TM protein structure (Marinko et al., 2019). While eukaryotes have multiple membranous organelles, they all lack measurable potential gradients, with the exception of the vacuole, whose proton pump maintains a positive inside-to-outside potential, rendering it topologically outside by prokaryotic criteria. Yet, the insertion of TM proteins into eukaryotic cell membranes still follows the PIR (Harley et al., 1998; Baker et al., 2017). This is an enigma.

## ER translocon: Sec61

The Sec61 translocon provides the sole TM channel for protein secretion and the major channel for TM protein insertion (Hegde and Keenan, 2022; Smalinskaite et al., 2022). Sec61 evolved from the SecY complex in bacteria and Archaea (Lewis and Hegde, 2021) and exists in all cells in several functionally differing complexes (Aviram and Schuldiner, 2017). The SRP recognizes the short N-terminal, seven-to-nine residue signal peptides in secreted proteins, if sufficiently hydrophobic, and the much longer first TMD of TM proteins as they exit the ribosome, if they are not too far from the N-terminus (Halic et al., 2004). The binding of the SRP to its receptor displaces the SRP and allows the signal peptide, or TMD, to enter Sec61, initiating ribosome-driven translocation. Signal peptide recognition and characteristics were recently reviewed (Nielsen et al., 2019). Disease-associated mutations in signal peptides cause failure in SRP interaction, leading to translation failure and mRNA decay (Tikhonova et al., 2019). Charges on extra-membranous loops of Sec61 confer a modest PIR response on entry (Goder et al., 2004), presumably responsible for the PIR response at Sec61 not subsequently provided by Ste24 and Spf1. If the TMD is too far from the N-terminus or if a signal peptide’s hydrophobicity is weak, post-translational protein insertion occurs using alternate chaperone complexes (Osborne et al., 2005; Aviram and Schuldiner, 2017).

## Single-pass and multi-pass TM proteins

Approximately 45% of all TM proteins have a single TMD (single-pass), and these fall into several classes (Hegde and Keenan, 2022): Type 1 proteins have a signal peptide, and their TMDs exit Sec61 via the lateral gate; after signal peptide removal, they are inserted Nexo as for secreted proteins (Hegde and Kenan, 2022). In Type 2, the TMD is inserted Ncyt. The TMD of Type 3, with N-termini no longer than 50 residues, is inserted Nexo (Aviram et al., 2017), usually by the EMC complex (Chitwood and Hegde, 2019). All types are potential substrates for the PIR quality control system. SRP binding has a broad affinity range that the ER insertases must accommodate, requiring multiple, often functionally overlapping insertion mechanisms (Aviram and Schuldiner, 2017; Hegde and Keenan, 2022).

All TM classes can be multi-pass; the topology of their TMDs alternates and is determined by that of the first TMD, TMD1. After insertion, the TMD1 of a multi-pass TM protein is translocated to an insertase complex that is associated with, but separate from, Sec61 for pairwise insertion of subsequent TMDs by the GEL complex (Smalinskaite et al., 2022). Presumably, these multi-pass proteins can only be monitored by the PIR quality control system before additional TMDs are added, as the Spf1 and Ste24 cavities cannot internalize or accommodate multi-pass TM proteins. Monitoring of TMD1 topology during translocation may be particularly important as inverted multi-pass TMD1 insertion would impair subsequent TMD insertion, requiring correction, probably by ERAD.

### The EMC, GET, and GEL insertases: Oxa1 family members

The ER membrane protein complex (EMC) was discovered in a screen of a yeast deletion library (Giaver et al., 2002) for genes whose deletion resulted in the accumulation of misfolded proteins in the ER lumen, leading to activation of the Hac1p transcription factor and expression of UPR genes (Jonikas et al., 2009). Components of the GET pathway (guided entry of tail-anchored proteins) were first described by Schuldiner et al. (2005). The Sec61, EMC, and GET insertase complexes, together with variants of the Sec61 complex and the GEL complex for polytopic TM protein insertion, comprise the ER TM protein insertases. The EMC and GET insertases are more efficient than Sec61 for the insertion of specific substrate classes, but the loss of neither pathway is lethal, so insertases are selective but not exclusive. The EMC, GET, and GEL insertases are members of the Oxa1 family. Oxa1 insertase (Lurink et al., 2001). Translocation often results in Nexo insertion, though Cexo insertion can also occur (Chitwood et al., 2018; Wu and Rapoport, 2018; Wu et al., 2020).

The TM components of the EMC are encoded by a set of six–seven genes, depending on the species, whose products can be immunoprecipitated as a stoichiometric TM protein complex (Jonikas et al., 2009; Chitwood and Hegde, 2019). The structure of this complex was described by Pleiner et al. (2020). Spf1 is commonly associated with the EMC (Ast et al., 2016). TM domains exiting the ribosome at the ER have a transient opportunity to dock at the EMC before docking at Sec61 (Chitwood et al., 2019; Hegde and Kenan, 2022). The EMC Oxa1 insertase (EMC3) prefers TM domains of lower hydrophobicity than does Sec61, but the functions of the EMC and Sec61 overlap. In G protein-coupled receptors (GPCRs), negatively charged N-terminal peptides preceding TMD1 will insert in the EMC vestibule and be translocated to Nexo (Chitwood and Hegde, 2019), obeying the PIR. TMD1 is then translocated to the PAT insertase complex associated with Sec61; the subsequent six TMDs are inserted pairwise after transfer to the PAT-associated GEL complex (Smalinskaite et al., 2022). The role of the EMC in the insertion of the β1 adrenergic receptor (β1AR, a GPCR) was studied in human rough microsomes (Chitwood et al., 2018). Nexo insertion with correct topology and folding was efficient, as shown by the binding of alprenolol, a β1AR inhibitor. In *EMC5Δ* cells, in which the EMC fails to assemble, default insertion at Sec61 resulted in about 50% β1AR destruction, presumably a result of high rates of topologically erroneous Cexo insertion. Correspondingly, alprenolol binding in EMC5Δ rough microsomes also decreased by 50%, presumably due to non-functional Cexo insertion at Sec61. In a construct that contained only β1AR TMD1 and its following short cytosolic loop, *in vitro* insertion was Nexo. In the absence of the EMC, insertion was ∼50% Cexo, again illustrating the high error rate of default insertion at Sec61.

## GET insertion of tail-anchored proteins

The GET pathway inserts tail-anchored proteins with strongly hydrophobic TMDs and proteins with similarly hydrophobic GPI C-terminal anchors, presenting them post-translationally for import at the GET1–GET3 complex (Schuldiner et al., 2008; Ast et al., 2016), another Oxa1 family insertase. The EMC plays a major role as an alternate to the GET pathway for tail-anchored TM protein insertion and post-translational secretion of proteins whose signals do not efficiently associate with the SRP (Baum and Baum, 2014; Wang et al., 2014; Aviram et al., 2017). Cytosolic aggregation is prevented by the binding of abundant chaperones such as HSP70s, eventually guiding export at Sec61 using ER luminal Kar2/Bip for ATP-driven ratcheted entry (Jung et al., 2013; Dederer et al., 2021).

## Selection for yeast mutants unresponsive to PIR signals; the PIR-quality control system

Yeast mutants unresponsive to PIR signals were selected to discover the mechanisms for response to TMD-adjacent peptide charge signals (Tipper and Harley, 2002). At that time, Sec61 was the only established translocon; the fascinating complexity of TM protein insertion is now apparent (Smalinskaite et al., 2022). Insertion of the model TM proteins used in mutant selection involves the EMC in addition to Sec61; our data, however, demonstrate that this is irrelevant since the major determinant of the PIR is a post-insertion quality control system independent of the insertion mechanism.

**FIGURE 2:**
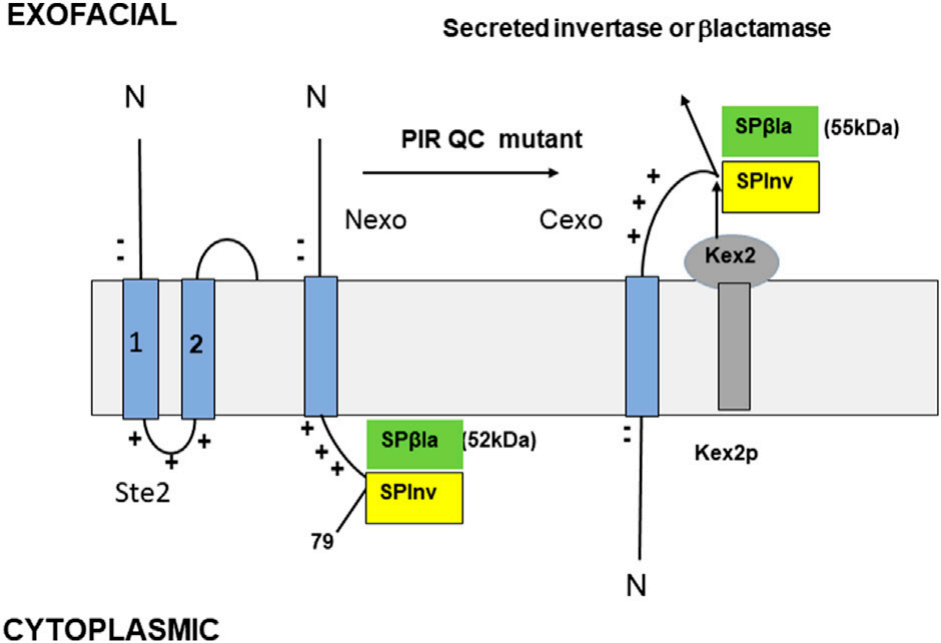
Model TM proteins (Tipper and Harley, 2002). The N-terminus of Ste2 (left) is shown with only TMDs 1 and 2. Two N-terminal negative charges and three C-terminal positive charges are all within the eight residues of TMD1, resulting in a charge difference of +5. The SPβla and SPInv constructs are shown in Nexo (center) and Cexo (right) topology. S is the N-terminal 79 residues of Ste2, including its Nexo TMD1 and the first cytoplasmic loop with a single exofacial N-glycosylation site; P is a peptide with two glycosylation sites that are cleaved by Kex2 in the Golgi if the fusion is Cexo. The gel mobilities of the Nexo and Cexo forms of SPβla are 52 and 55 kDa, respectively; their ratio measures the SPβla topology (% Cexo). The SPInv fusion is used for PIR mutant selection; when Cexo is inserted, Golgi cleavage and secretion are efficient, allowing invertase secretion and growth on sucrose plates. An assay of secreted invertase activity, compared to a control construct with almost complete Cexo insertion provides an independent assay of topology.

The Sec61-secreted *SUC2* gene product, invertase (Inv), cleaves sucrose into glucose and fructose. Yeast lacks a sucrose transporter, so invertase is essential for growth in sucrose media. *SUC2* was expressed via a Golgi-cleavable fusion to the C-terminus of a TM protein with a strong PIR signal directing Nexo insertion. In a *SUC2Δ* strain, Nexo insertion of the fusion would prevent invertase secretion (Figure 2). Growth on sucrose plates should then be selected for the desired mutations. The TM protein chosen was S (Figure 2), a 79-residue N-terminal fragment of Ste2; the GPCR receptor for the yeast α-mating pheromone encompassing its 50-residue N-terminus, TMD1, and the 8-residue following cytoplasmic loop. S is very similar to the β1AR fragment described by Chitwood et al. (2018). The charge difference across TMD1 is strong (+5, Figure 2). A ß-lactamase (ßla) fusion to TMD1 is inserted Nexo (Harley and Tipper, 1996). P is a doubly N-glycosylated fragment of the K1 killer preprotoxin, cleaved in the Golgi by the Kex2 protease (Bostian et al., 1980). The fusion product is SPInv (Tipper and Harley, 2002; Figure 2). Cexo insertion should result in invertase secretion. Insertion topology was determined by the secreted invertase activity. SPInv expression resulted in just 3% of the secreted invertase activity of the *SUC2* parent strain (Tipper and Harley, 2002), which is insufficient for growth on sucrose as this requires sufficient invertase production to form cell wall-bound aggregates. In contrast, a SPInv fusion with a −4 charge difference, predicted to insert Cexo, expressed 95% of the parental strain invertase activity, so it was inserted 95% Cexo and is efficiently cleaved in the Golgi (Tipper and Harley, 2002).

Topology was independently determined by the expression of C-terminal ßla fusion to SP (SPßla, Figure 2). SPßla topology is measured by the ratio of 52 kDa (Nexo) and 55 kDa (Cexo) forms (Figure 2); after pulse-labelling and immunoprecipitation. In normal cells, SPßla was inserted with 3% Cexo, as for SPInv (Tipper and Harley, 2002). *SUC2Δ* cells expressing SPInv were selected for growth on sucrose after severe ethyl methanesulfonate-induced or transposon insertion mutagenesis. Mutants allowing strong growth were detected at very low frequencies in only two genes, *SPF1* and *STE24.* Deletion mutants had the same phenotype as the strongest selected mutants; *SPF1Δ* and *STE24Δ* strains produced 22% and 24%, respectively, of the *SUC2* parent strain invertase activity. These data were corroborated by pulse-labelled analysis of SPßla insertion (Figure 2); in the *SPF1Δ* and *STE24Δ* strains, insertion was 22% and 25% Cexo, respectively (Tipper and Harley, 2002). Since complete loss of PIR topology control should result in a 50/50 Cexo/Nexo ratio, about half of this control is lost in either deletion mutant. A double mutant strain was highly stress-sensitive, was both cold- and heat-sensitive, and had 38% of parent strain invertase activity, indicating a 38/50 or about 76% loss of topology control; substrate recognition by Spf1 and Ste24 appears to overlap about 50%, but their other substrates are not shared, so effects of deletion are partially additive (Tipper and Harley, 2002).

### Nexo SPInv insertion occurs at the EMC, and Cexo insertion occurs at Sec61

Nexo forms of SPInv and SPßla fusions have 50-residue N-terminal peptides flanking the TMD (Figure 2) and, thus, can translocate at the EMC, as described for the TMD1 of other GPCRs (Chitwood et al., 2018). If insertion were Cexo, however, the bulky C-terminus (Figure 2) would preclude insertion into the EMC3 Oxa1 vestibule, so insertion would necessarily occur at Sec61. The residual bias of approximately 24% toward correct topology in the double mutant would then reflect weak selection at the Sec61 translocon (Goder et al., 2004). In our hypothetical PIR quality control model, Spf1 and Ste24, by eliminating most of the positive external Cexo inserts, are the major effectors of the PIR for single-pass TM proteins, and also for multipass TM proteins if topology of TMD1 is effectively monitored. Spf1 and Ste24 are conserved in all eukaryotes and so must have evolved as this cell type evolved to minimize OMM protein insertion at the ER (an exclusive function of Spf1) and dysfunction caused by highly error-prone TM protein insertion at the ER (a function of both Spf1 and Ste24). Spf1, by dislocating Cexo inserts (McKenna et al., 2020; McKenna et al., 2022), allows reinsertion, a frugal, energy-conserving mechanism; Ste24, by contrast, presumably imposes a major strain on cell resources by fragmenting incorrectly inserted TM proteins for proteasomal disassembly (Figure 1).

## Role of Spf1 in eliminating unfolded proteins from the ER

Spf1 deletion in yeast causes synthetic lethality with the deletion of Hac1 (Cronin et al., 2002), the transcription factor that induces the UPR (Ron and Walter, 2007). The comprehensive yeast deletion library (Giaver et al., 2002) was screened for deletions causing induction of the UPR (Jonikas et al., 2009); the range was 2–18 fold. The strongest were *SCJ1*, a DnaJ chaperone homolog that interacts with Kar2/Bip (Jung et al., 2013) for post-translational import to the ER lumen, and *ARV1*Δ, essential for glycophosphatidylinositol (GPI) anchor synthesis. Strong induction of the UPR is predictable for both deletions. The next strongest inducers were *SPF1*Δ and *STE24*Δ, both of which caused 12–13 fold induction, higher than BST1 (adenyl cyclase 2) orLAS21 (also equired for GPI anchor synthesis), and a full 50% higher than any of the remaining deletions, such as ERAD, EMC and GET component deletions, none of which caused more than 8-fold induction (Jonikas et al., 2009). The similarly massive accumulation of unfolded proteins in the ER lumen caused by either *SPF1*Δ or *STE24*Δ is consistent with the existence of many substrates for their quality control activities, estimated to be one third of the 33% average proteome turnover (Schubert et al., 2000), and with the massive induction of Hac1 expression caused by their deletion (Jonikas et al., 2009).

## Spf1 and OMM protein insertion

Spf1 is a P5A-ATPase, a transmembrane helix dislocase that is particularly important for expelling nuclear-encoded tail-anchored OMM proteins erroneously inserted at the ER (McKenna et al., 2020) for translocation to the OMM, which is their proper location. These OMM proteins are inserted post-translationally as they have a single, relatively short, C-terminus-proximal TMD of relatively low hydrophobicity; the following short C-termini contain several positive charges (Chio et al., 2017). These proteins bind abundant chaperones such as HSP70 family members and calmodulin after exiting the ribosome. There are no known chaperones for specific targeting to the OMM, the default location for insertion (Hegde and Kenan, 2022). Their C-terminus inserts Cexo into the mitochondrial inter-membrane space (Krumpe et al., 2012). However, nearly half insert Cexo at the ER (McKenna et al., 2020); this brings their C-terminal positive charges into the ER lumen, contrary to the PIR. Import of OMP25, a yeast OMM protein containing a UV-activated cross-linker tag in its TMD, into yeast membrane vesicles identified several interacting OMM proteins but only a single and prominent ER interactor, Spf1 (McKenna et al., 2020). Spf1 is the only P5A-ATPase in yeast, and its human homolog, Atp13A1, is the sole P5A-ATPase in human cells. P-type ATPase families were reviewed by Andersen et al. (2016). Closely related homologs of Spf1 exist in all eukaryotes. In human rough-ER microsomes, Atp13A1 dislocated OMP25, but only when Atp13A1 retained its ATPase activity and an ATP source was present, demonstrating that the ATPase-dependent conformational shift of Atp13A1 is essential for the dislocation of the mislocalized OMM protein to the cytosol (McKenna et al., 2020). The mitochondrial OMM protein Msp1 performs an analogous function by dislocating mislocalized ER proteins from the OMM for ER insertion (Matsumoto et al., 2019).

**FIGURE 3:**
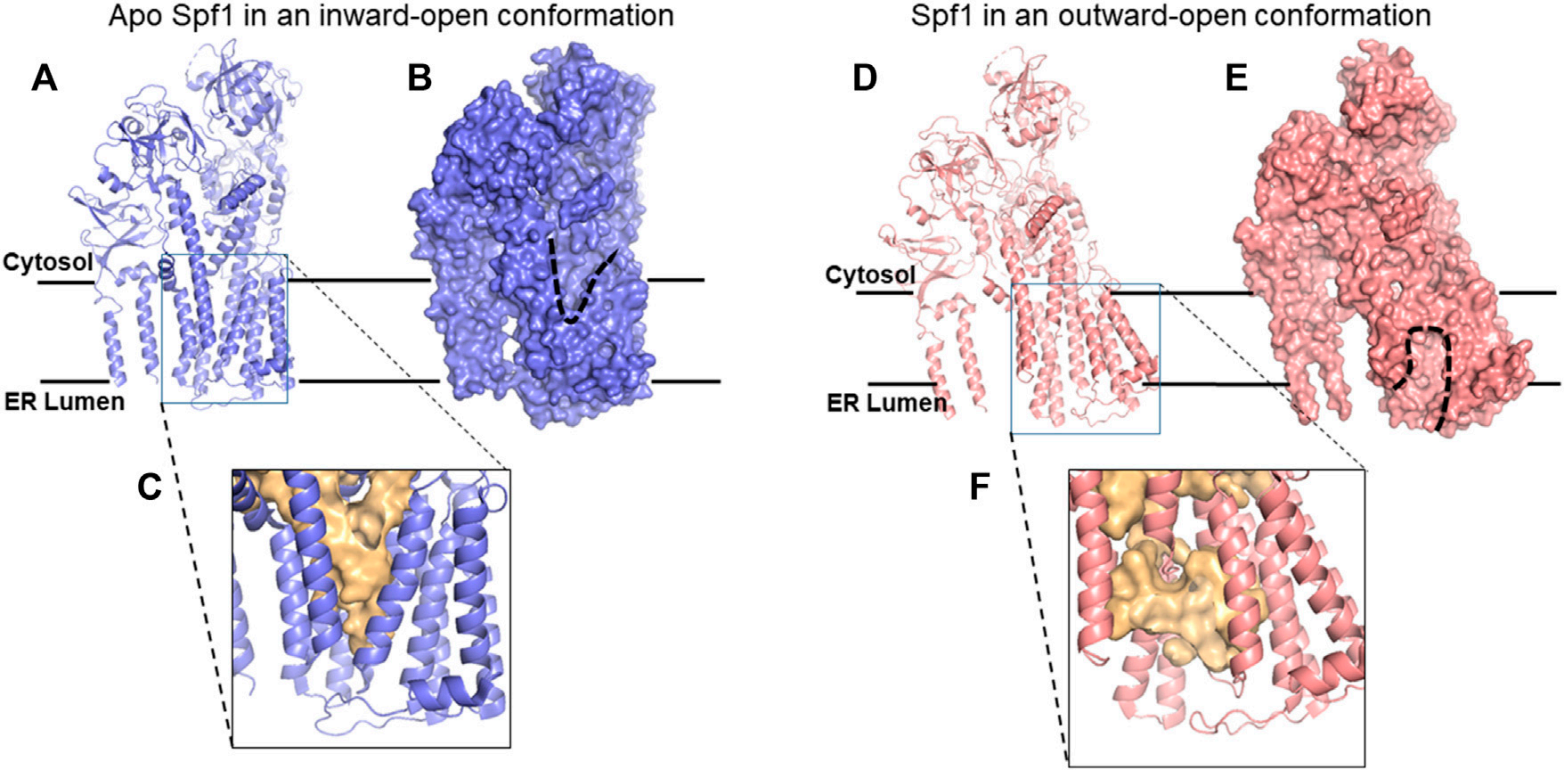
Cryo-EM structures of Spf1. **(A)** Ribbon representation of apo Spf1 in a conformation open to the cytosol (inward). **(B)** Surface representation with the “V”-shaped substrate-binding pocket outlined (dashed line); bound substrates can exit the cytosol. **(C)** Enlarged view of the substrate-binding pocket (light gold surface) with a closed luminal gate. **(D)** Ribbon representation of BeF3-bound Spf1 in a conformation open to the lumen (outward). **(E)** Surface representation with the substrate-binding pocket outlined (dashed line). **(F)**Enlarged view of the substrate-binding pocket (light gold surface) with an open luminal gate. This cavity should preferentially bind ER-TM proteins with positively charged luminal peptides. Reproduced with permission from McKenna et al. (2020).

As shown in Figure 3, seven of Spf1’s 10 TM helices enclose an unusually large, membrane-spanning, water-filled cavity. Conserved kinks in TM helices keep fenestrations open to the lipid bilayer in either conformation, potentially allowing TM protein substrate entry. The conformation with a binding pocket open to the lumen (Figure 3) allows positively charged luminal Nexo peptides to lead TM protein substrates into Spf1’s cavity, presumably guided by interaction with negative charges on the cavity’s inner surface (McKenna et al., 2020; McKenna et al., 2022). Internalized substrates presumably rearrange rapidly to an energetically minimal state with a hydrophobic core exposing hydrophilic residues for interaction with the cavity’s inner surface. ATP hydrolysis results in substrate exit to the cytosol (Figure 3), causing substrate dislocation. In the conformation that is open to the lumen (Figure 3), the cavity contains an additional helical component (McKenna et al., 2020), which apparently represents an averaged image of an internalized single-pass TM protein substrate; this is not visible in Figure 3, as the relevant data were described but not published (McKenna et al., 2020; McKenna et al., 2022). Type P4 ATPases, the closest relatives of type 5, transport lipids (Palmgren and Nissen, 2011), the first report of P-type ATPases transporting substrates other than ions. P4 ATPases were later called lipid flippases (Andersen et al., 2016). The human P5B-ATPase, Atp13A2, is a lysosomal polyamine transporter (van Veen et al., 2020), a secondary macromolecule transporter. Spf1/ATP13A1 is a third example, demonstrated to dislocate single-pass TM proteins, a novel, and important observation (McKenna et al., 2020; McKenna et al., 2022). An Spf1/ATP13A1-dislocated OMM protein presumably has repeated chances for insertion in its appropriate OMM location or to re-enter the ER. In the absence of Spf1/Atp13A1, OMM proteins inserted at the ER are hydrolyzed by signal peptide protease (SPP), a member of the ancient family of aspartyl proteases with active sites buried in the membrane; these include the presenilins, the active components of γ−secretase (Weihofen et al., 2002; Yucel and Lemberg, 2020). The fragments they produce are delivered to the proteasome by ERAD after ubiquitination (Dederer et al., 2020; McKenna et al., 2022). Ste24 does not recognize these misplaced OMM proteins, allowing Spf1 to maximize its energy-conserving relocation of OMM proteins to the OMM. SPP also cleaves other tail-anchored TM proteins in the ER, such as HO-1 (heme oxygenase), a component of squalene synthase, and a few other tail-anchored TM protein substrates (Boname et al., 2014; Yucel and Lemberg, 2020; McKenna et al., 2022). We propose that Ste24 recognizes and fragments many of the single-pass TM proteins erroneously inserted at the ER in positive outside topology, including about half of those recognized by Spf1 and about half of those not recognized by Spf1 (Tipper and Harley, 2002). We propose that the PIR quality control system complements the multiple mechanisms for eliminating misfolded ER TM proteins (Chen et al., 2023). The major roles played by Spf1 and Ste24 are illustrated by their dominant roles in eliminating misfolded ER proteins (Jonikas et al., 2009).

In *SPF1*Δ cells, mitochondrial OMM proteins were not only massively mislocalized to the ER, but they were also markedly depleted from the mitochondria, illustrating the major role of Spf1 in maintaining normal ER-OMM OMM protein distribution (Krumpe et al., 2012). Sterol content is much lower in the OMM than in the ER, and OMM TA proteins have a strong preference for *in vitro* insertion into membranes of low sterol content (Krumpe et al., 2012). In *SPF1Δ* cells, the ergosterol content of the OMM and the ER is similar (Krumpe et al., 2012), suggesting that OMM protein content controls ergosterol content, which is more probable than control of protein distribution by ergosterol content ((Krumpe et al., 2012). Ste24 does not contribute to the control of OMM protein localization, which is entirely dependent on Spf1 or SPP.

## Ste24 and ZMPSte24: transmembrane zinc metalloproteases

Yeast *STE24* encodes a TM zinc metalloprotease (ZMP), required for processing the precursor of the yeast’s mating pheromone (Tam et al., 1998), and since this precursor contains a C-terminal isoprenylated CAAX box, Ste24 was presumed to cleave these C-termini. However, this was incorrect; Ste24 cuts the a-factor precursor at other sites (Goblirsch and Wiener, 2020). The yeast CAAX box peptidases have no direct role in PIR responses (Tipper and Harley, 2002). The human homolog, ZMPSte24, can restore Ste24 function in *STE24Δ* yeast cells, demonstrating functional conservation (Ast et al., 2016). Ste24 structure was determined by Pryor et al. (2013). Ste24 evolved from prokaryotic TM ZMP ancestors such as HtpX of *E. coli* (Arolas et al., 2014). Comparison of the structures of the yeast *Saccharomyces mikatae* Ste24 and ZMPSte24 showed strict conservation over this huge evolutionary gap, with an RMSD (relative mean squared deviation) for amino acid α carbon positions of only 1.7 Å (Goblirsch et al., 2019). Sequences among a group of 58 Ste24 orthologs covering widely divergent species contain 38 invariant residues and 30% similarity, so not only are they highly conserved, but they all share the same structure (Goblirsch et al., 2019). Ste24 has multiple specific substrates, such as prelamin A in animals, which is processed into lamin A at the inner nuclear membrane. Lamin A then assembles into the nuclear lamina, which provides mechanical stability to the nuclear envelope, maintaining nuclear architecture and controlling gene expression dependent on that architecture (Smith et al., 2021).

## Ste24 structure

**FIGURE 4:**
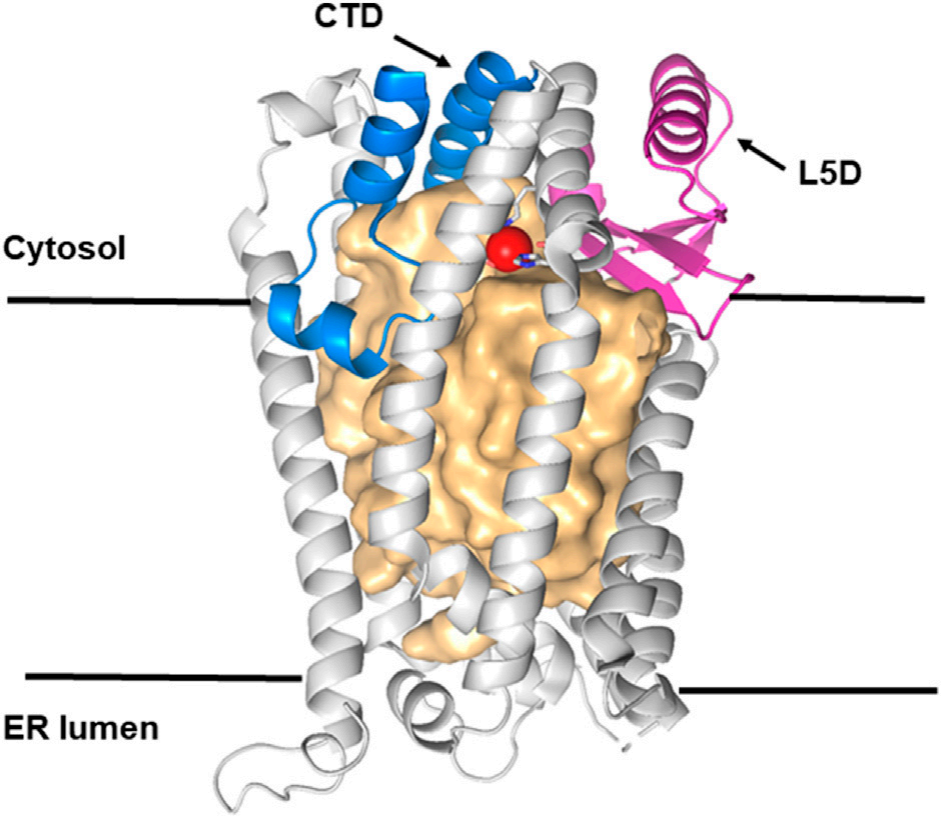
Structure of Ste24p. Ribbon representation of the ß barrel structure of Ste24p (light gray) with a large central cavity of more than 12,000 Å^3^ represented as a light gold surface. Ste24p is a membrane-bound zinc metalloprotease (ZMP) with seven TM α-helices. Helices VI and VII contain the zinc-binding site (Zn shown as a red sphere). Membrane–cytosol interface domains are shown in magenta (L5D, the loop 5 domain) and in blue (the C-terminal section of the ZMP domain). Reproduced with permission from Goblirsch and Wiener (2020).

The structure of ZMPSte24 (Pryor et al., 2013) can be divided into three domains (Goblirsch and Wiener, 2020; Goblirsch et al., 2019; Figure 4): its ZMP protease structure lies at the cytosolic interface and is similar to that of soluble ZMP proteases such as thermolysins; it comprises 79 residues, with 18 (23%) completely conserved among the 58 Ste24 orthologs; the adjacent L5D domain also lies at the cytosolic interface and contributes a β-strand to the ZMP active site. It consists of 135 residues, with 13 (10%) completely conserved (Goblirsch et al., 2020) (Figure 4). The unique α-barrel TM domain contains 247 residues; although only seven of these are completely conserved, these include the aromatic residues that mark the termini of specific TM helices and three proline residues that introduce kinks in these helices (Goblirsch et al., 2020). This implies that conservation of the precise α-barrel shape, with its several fenestrations, is essential for function, potentially allowing access to TM-protein substrates from the surrounding membrane, as for Spf1. The internal, roughly cylindrical cavity of Ste24 is very large (12,000 (23^3^) Å^3^), water-filled, and lined with relatively hydrophilic residues, many of which are negatively charged. Some of these are located close to the L5D domain (Figure 4) (Goblirsch and Wiener, 2020) and they include the fully conserved aspartate 164. Determining the effect of a D164T or a potentially more disruptive D164K mutation on Ste24 function could prove informative; substrate selection might be significantly altered.

Partially reduced function in ZMPSte24 causes lipodystrophies (maldistribution of lipid tissue; Guénantin et al., 2014). Lipodystrophy is potentially a consequence of the treatment of HIV patients with inhibitors of the HIV aspartyl protease since ZMPSte24 may be sensitive to these inhibitors (Goblirsch and Wiener, 2020), but this is not established. Mutations that cause severe reductions in ZMPSte24 activity cause laminopathies, such as progerias, as do lamin A mutations (Ahn et al., 2022). Progeria severity in ZMPSTE24 mutants correlates inversely with the residual Ste24 proteolytic activity (Goblirsch and Wiener, 2020).

## Roles of Ste24 in relieving clogged insertases and in type 2 diabetes

Proteins that fail to bind the SRP because they are too short, their TMD is too far beyond the N-terminus, or their signal peptides have relatively low hydrophobicity and are secreted post-translationally. Secretion requires the Kar2 (yeast) or Bip (metazoan) ER luminal chaperone to bind these substrates and, by its DNAj-stimulated ATPase action, ratchet them into the ER lumen using a distinct Sec62 translocon complex (Johnson et al., 2013; Pobre and Hendershot, 2019; Runnebohm et al., 2020). Substrate contact with Kar2 causes attached chaperones to dissociate from C-terminal domains; these are then prone to fold before insertion, clogging the insertase. Ste24 clears these clogs (Ast et al., 2016), functionally overlapping with Hrd1 ubiquitin ligase and ERAD for clearance (Runnebohm et al., 2020). Clog cleavage by Ste24 requires substrate withdrawal, apparently using the Dfm1-recruited AAA ATPase Cdc48 (Dederer et al., 2020), since a Dfm1 deletion prevents unclogging (Ast et al., 2016). Fragments of clogged substrates or other Ste24 substrates would be degraded by the proteasome. Dfm1 functions were studied by Sato and Hampton (2006). Effects of a *DFM1Δ* mutation or expression in a CDC48^ts^ strain (Ast et al., 2016) would test the potential role of this withdrawal mechanism in Ste24 function. The hexameric Cdc48 structure was described by Bodnar et al. (2018).

Type 2 diabetes causes pancreatic β-cell failure and is a burgeoning worldwide scourge. In patients with the disease, islet amyloid polypeptide (IAPP), a 37-amino acid hormone that is co-secreted with insulin, misfolds and clogs the translocon secretion channel, contributing to β-cell failure and causing the formation of amyloid deposits in pancreatic islets (Clark et al., 1988). Mouse IAPP does not form amyloid, but mice overexpressing human IAPP develop spontaneous type 2 diabetes (Kayatekin et al., 2018). Expression of a human IAPP hexamer (6xIAPP), designed to accelerate amyloid formation, accelerated type 2 diabetes development in mice. In yeast cells, inducible expression of 6xIAPP caused a strong growth defect, and the 6xIAPP oligomer accumulated in the secretory pathway and the cytoplasm, consistent with clog formation, providing a model for IAPP toxicity (Kayatekin et al., 2018). Overexpression of Ste24 suppressed 6xIAPP toxicity, whereas deletion of Ste24 exacerbated it; expression of ZMPSte24 suppressed 6xIAPP toxicity in a *STE24Δ* mutant, illustrating a conserved protease function in clog destruction; expression of Ste24 protected rat insulinoma cells against toxicity mediated by 6xIAPP expression (Ast et al., 2016; Kayatekin et al., 2018). Deletion of Ste24 or Dfm1 enhanced 6xIAPP toxicity in yeast (Kayatekin et al., 2018), consistent with essential roles for Dfm1 and Cdc48 in IAPP clog extraction by Ste24, although direct interaction was not demonstrated (Kayatekin et al., 2018; Dederer et al., 2021). The mechanism of access for the withdrawn clog to the Ste24 ZMP active site is unknown.

## Ste24 and control of secretion from the cytosol

*Cpy*, a yeast glycopeptide protease, is secreted to the vacuole post-translationally. After insertion at Sec61 and glycosylation in the Golgi, pro-Cpy normally transits to the vacuole, where cleavage by Pep4 completes maturation. If the *Cpy* signal peptide is completely deleted, about 65% accumulates in un-glycosylated form in the cytoplasm, although 35% still reaches the vacuole in fully mature form (Blachly-Dyson and Stevens, 1987; Hosomi et al., 2020). This increases to 95% in *STE24*Δ cells. Pep4ΔN23, also lacking its signal peptide, behaves similarly; 23% reaches the vacuole in WT cells and >90% in *STE24Δ* cells. Ste24, thus, recognizes and eliminates the aberrant secretion of Cpy and Pep4 from the cytosol. Ste24 recruitment would presumably require recognition of such aberrant secretion substrates and their withdrawal from Sec61, potentially involving Dfm1 and Cdc48, as for insertase clog clearance. Preventing these non-functional secretion events may be a significant part of Ste24’s quality control functions.

## Roles of Spf1 and Ste24 in eliminating TM proteins inserted in the ER contrary to the PIR

While Spf1 and Ste24 have very different effector mechanisms, both eliminate TM proteins inserted in an inverted topology contrary to the PIR with similar efficiency (Tipper and Harley, 2002). Both have large, membrane-spanning, water-filled cavities and while Ste24’s cavity is considerably larger than that of Spf1, they have basic structural similarities specifically related to the demonstrated substrate recognition and entry functions of Spf1 (McKenna et al., 2020). Both cavities are enclosed by seven TM helices with conserved kinks that provide stable fenestrations, potentially allowing TM protein entry and both internal surfaces have distinctly negative electrostatic potentials (Figures 3, 4), consistent with similar mechanisms for substrate recognition. OMM proteins are tail-anchored TM proteins recognized in the ER only by Spf1, while about half of other Spf1 and Ste24 substrates overlap (Tipper and Harley, 2002), so substrate recognition mechanisms must also partially overlap. Mechanisms following recognition are unrelated; for Spf1, ATP hydrolysis results in a series of conformational shifts between the E2 state, open to the lumen, and the E1 state, open to the cytosol (Figure 3) (McKenna et al., 2020). In the E2 conformation, electronegative potential in the cavity presumably promotes entry of the short, positively charged luminal substrate peptides, guiding lateral entry of the entire TM proteins via fenestrations. Ste24 ZMP activity is required for the PIR quality control function (Tipper and Harley, 2002); internalized substrates presumably rearrange to an energetically minimal state, as for Spf1. This might allow diffusion to Ste24’s cytosolic face and interaction with the L5D and ZMP modules; however, efficient hydrolysis may require an external motive force. We hypothesize that this may be provided by Dfm1-recruited Cdc48 AAA ATPase (Dederer et al., 2021) and insertase clog removal. Substrate fragments would be pulled into the cytosol for delivery to the proteasome (Figure 1). However, no direct evidence demonstrates substrate entry into the Ste24 cavity, subsequent internal access to the ZMP site, cdc48 withdrawal, or proteasomal destruction.

## The PIR quality control system

The proposed PIR quality control system, consisting of the effector pairs Spf1/Ste24 (yeast) and *ATP13A1/ZMPSte24* (human), presumably evolved along with the eukaryotic cell to address the cellular stress caused by mitochondrial OMM proteins inserted into the ER (Spf1-specific substrates) and the massive accumulation of inverted TM proteins in the ER resulting from high rates of aberrant ER insertion. The PIR quality control system supplements ERAD, but with specific recognition and substrate criteria; substrates are recognized by their defining positive outside ER luminal peptide inserts; the resulting correction imposes the PIR. The positive outside topology of PIR quality control substrates is required for entry into the Spf1’s cavity (McKenna et al., 2020, McKenna et al., 2022) and presumably for the proposed entry into the larger Ste24’s cavity. The sharing of some substrates with ERAD (Runnebohm et al., 2020) is an example of normal redundancy in cellular functions. This PIR quality control system is highly effective but never 100% effective, as illustrated by the 97% Nexo insertion of the model SPInv and SPβla TM proteins with −4 PIR signals (Tipper and Harley, 2002). A weaker PIR response is apparent in a SPInv fusion with a +2 PIR signal, entirely due to the two TMD-adjacent N-terminal negative charges in S (Figure 2); insertion was 8% Nexo in WT cells, increasing to about 30% in *SPF1Δ* and *STE24Δ* cells and 43% in the double-mutant strain, so this higher rate of erroneous insertion is still effectively corrected by the PIR quality control system.

## ZMPSte24 in antiviral responses

ZMPSte24 plays an important role, apparently as a major effector in a broad-spectrum constitutive defense against fusion between the membranes of host cells and the membranes of viral pathogens such as influenza, coronaviruses, HIV, Ebola, and Zika (Brass et al., 2009; Narayana et al., 2015; Fu et al., 2017; Majdoul et al., 2022). Ste24 is also effective against SARS-Cov-2 infection (Shilagardi et al., 2022). Screening for genes affecting γ-interferon-induced responses to influenza revealed a role for the inducible transmembrane protein (IFITM) family (Brass et al., 2009). ZMPSte24 interacts with IFITM3 (Fu et al., 2017), the best-studied and most active family member, which may chaperone ZMPSte24 to its viral fusion targets. Antiviral activity did not require ZMPSte24 protease activity but may result from the recognition of virus–host cell membrane hemifusions (Hosomi et al., 2020). Disruption of trafficking is suggested to cause the transit of virus-infected endosomes to the vacuole for destruction, but the mechanisms remain conjectural (Majdoul et al., 2022).

## Identification of “constitutive” PIR quality control substrates by affinity labeling

Recent progress toward identifying Atp13A1 and ZMPSte24 substrates comes from the use of proximity labeling techniques in which a protein bait, coupled to a promiscuous form of biotin ligase, is used to tag transient protein interactants within a range of ∼10 nm (average protein size) for their subsequent identification (Go et al., 2021). A total of 192 proteins or peptides, representing all internal cellular compartments of human HEK293 cells, were converted into bait. These were then expressed in HEK293 cells. The proteins identified as interacting with Atp13A1 are presumably frequently contacted and are essentially constitutive cellular targets for dislocation; these included a particular signal peptide fragment, several ER TM proteins, TM proteins in an ER/Golgi intermediate compartment, subunits of the ER Ca^2+^ channel, components of a lipid droplet export compartment, and Sec62 and Sec61B (Go et al., 2021). All, except the signal peptide, are potential orphan (excess) components of major ER TM protein complexes, which are plausible Atp13A1 substrates. Other interactants were components of endosomes, the plasma membrane, and nuclear pores, perhaps also orphans or products of trafficking errors (Go et al., 2021). None of the identified interactants had a plausible role in substrate recognition (Go et al., 2021).

For ZMPSte24, the conclusions were similar (Go et al., 2021). Approximately 40% of the interactants were shared with Atp13A1, consistent with the quantitative results of Tipper and Harley (2002), showing substantial but incomplete substrate overlap in the PIR quality control. The remaining 60% are likely to represent orphan components of TM protein complexes, or proteins that failed to transit to their proper destinations; these included ER luminal proteins, mitochondrial matrix proteins, and an OMM protein that is not an identified Spf1 substrate (Go et al., 2021). As is probably true for dislocation by Atp13A1, ZMPSte24 hydrolysis of random products of aberrant TM protein insertion or post-translational secretion probably far exceeds hydrolysis of the constitutive substrates identified by proximity labeling (Go et al., 2021). None of the identified Ste24 interactors had a plausible role in substrate recognition.

### The eukaryotic PIR quality control system: a hangover from prokaryotic ancestors?

Basic similarities in the structures of Spf1 and Ste24 are the only clue to a common mode of substrate selection, independent of the obvious differences in substrate removal mechanisms. The proposed shared mechanisms for substrate selection by recognition of TMD-adjacent positively charged luminal peptides may explain how this quality control system imposes approximately 75% of the PIR after error-prone insertion. The role of membrane lipid charge on PIR responses needs to be taken into account (Dowhan et al., 2019). The role of Ste24 in the PIR quality control mechanism requires considerable further investigation.

The question remains as to why the PIR is still relevant in a eukaryotic cell. Plausible prokaryotic progenitors of eukaryotic cells, such as the archaeal Asgard family (Gabaldon, 2021; Liu et al., 2021), still had a positive outside prokaryotic plasma membrane potential as the internal membrane complexity and trafficking machinery evolved. This increasing complexity probably resulted in an increasing frequency of TM protein insertion errors, driving the selection of quality control effectors capable of recognizing and eliminating nonfunctional TM proteins inserted “positive outside.” Such erroneous insertions were defined over many millennia in their prokaryotic ancestors by the PIR, the determinant of topological “right and wrong.” The acquisition of mitochondrial precursors, presumably from other cells, would have provided the energy supply necessary for this increasingly expensive cellular lifestyle, but after the OMM protein gene migrated to the nucleus, OMM proteins would have been included as a major source of additional PIR quality control substrates. It is possible that Spf1 evolved to address this specific issue, while Ste24 evolved much earlier to meet more general housekeeping problems. Thus, the persistence of dependence on the PIR for quality control of TM protein topology in eukaryotic descendants, as ER and cytoplasmic membrane functions diverged, could therefore be considered a hangover or relict from their ancient prokaryotic ancestors. A search we made of the Asgard family sequences for critical sequences of Spf1 (ATPase domain) or Ste24 (ZMP domain) did not prove informative.

ER-phagy is a system that removes ERAD-resistant misfolded proteins and aggregates from the ER for lysosomal destruction, apparently working in concert with ERAD and the UPR to control TM protein quality in the ER (Chen et al., 2023). The PIR quality control system is another important component essential for maintaining ER TM protein quality. Defects in the UPR, ERAD, and ER-phagy have been shown to play major roles in cancer and neurodegenerative diseases (Chen et al., 2023). Defects in PIR quality control may cause similar pathology.

## Conclusion

We propose that Spf1/Atp13A1 and Ste24/ZMPSte24 share a common mechanism for PIR quality control substrate recognition, although this has only been demonstrated for Spf1/Atp13A1 (McKenna et al., 2020) and many aspects of Ste24 function remain unresolved. This hypothesis is based on basic similarities in structures that are essential at least for Spf1/Atp13A1 function (McKenna et al., 2020, McKenna et al., 2022). Direct analysis of the mechanism of Ste24 is a top priority to test the PIR quality control hypothesis and evaluate its importance. Testing the potential role of the conserved Ste24 aspartate 164 in substrate cleavage should be straightforward. Analysis of the potential roles of Dfm1 and cdc48 in substrate removal from Ste24 may substantially clarify Ste24’s mechanism. Dfm1 is a candidate for the unidentified mutant with about half of Spf1’s effect isolated by Tipper and Harley (2002). To test whether Ste24 substrates do pass through the Ste24 cavity to reach its ZMP domain, a strategically located biotin ligase fusion (Fenech et al., 2023), perhaps linked to a cytosol-accessible L5D residue (Figure 4), may identify transiently adjacent effectors or substrate fragments. A Ste24 substrate with a suitably positioned UV-activated cross-linker (McKenna et al., 2020) may potentially identify both STE24-interacting effectors and substrates, perhaps identifying features that distinguish them from Spf1 substrates.
